# Comparison of the Changes in Corneal Endothelial Cells after Pars Plana and Anterior Chamber Ahmed Valve Implant

**DOI:** 10.1155/2015/486832

**Published:** 2015-01-28

**Authors:** Ji Won Seo, Jong Yeon Lee, Dong Heun Nam, Dae Yeong Lee

**Affiliations:** ^1^Department of Ophthalmology, Graduate School of Medicine, Gachon University, Incheon 405-760, Republic of Korea; ^2^Department of Ophthalmology, Gachon University, Gil Medical Center, Incheon 405-760, Republic of Korea

## Abstract

*Purpose*. To compare the changes in corneal endothelial cells after pars plana Ahmed glaucoma valve (AGV) implantation with those after the anterior chamber AGV implantation for refractory glaucoma. *Methods*. The medical records of 18 eyes with pars plana implantation of AGV (ppAGV) were reviewed retrospectively and were compared with 18 eyes with the anterior chamber AGV (acAGV) implant. The preoperative and postoperative endothelial cells, intraocular pressure (IOP), and postoperative complications during the follow-up in both groups were compared. *Results*. The average follow-up was 18 months. The postoperative endothelial cells in the ppAGV and acAGV groups were 2044 ± 303 and 1904 ± 324, respectively (*P* = 0.25). The average percentage decrease in the endothelial cells in the ppAGV and acAGV groups at 18 months was 12.5% and 18.4%, respectively, and showed significant difference between the 2 groups (*P* = 0.01). No difference in IOP control and the number of postoperative glaucoma medications was observed between the 2 groups. *Conclusions*. Endothelial cell damage in the ppAGV group for refractory glaucoma appeared to be lower than that in the acAGV group. Therefore, pars plana implantation of AGV may be preferred as it may have lower level of endothelial cell damage while maintaining similar level of IOP control.

## 1. Introduction

Glaucoma drainage device (GDD) is used in the management of refractory glaucoma. In a multicenter randomized clinical trial, tube shunt surgery had a higher success rate compared to trabeculectomy, with similar reductions in intraocular pressure and the need for supplemental glaucoma medications [1]. In recent years, some surgeons are using tube shunts or GDD as first-line surgery and forgoing standard trabeculectomy surgery.

Typically, the GDD is placed in the anterior chamber and acts to shunt aqueous fluid to an equatorial implant [2, 3]. However, the implant can be placed through pars plana in eyes with advanced glaucoma having secondary angle closure or angle neovascularization, corneal diseases, and other anterior chamber abnormalities [4–6].

Several studies have shown that pars plana vitrectomy with implantation of a drainage tube achieved a success rate comparable to that of an anterior chamber implant in refractory glaucoma [7–9].

The implantation of an Ahmed glaucoma valve (AGV) has been studied for the management of intractable glaucoma since first described in 1995 [10]. The AGV can control intraocular pressure (IOP) whether the tube is placed in the pars plana or in the anterior chamber [11, 12].

The principal long-term complication of anterior chamber insertion is corneal endothelial failure [13]. Some studies have reported that the endothelial cell density decreases progressively after AGV implantation in the anterior chamber [14–16]. Corneal endothelial cell loss after the pars plana insertion of an AGV was mild and comparable to the cell loss observed after simple cataract surgery [12]. Nevertheless, there are no reports comparing the endothelial damage of pars plana insertion and anterior chamber insertion of the AGV.

Therefore, the changes in corneal endothelial cells after pars plana implantation of an AGV implant with a vitrectomy were compared with those of the implantation of an AGV in the anterior chamber for the treatment of refractory glaucoma.

## 2. Methods

This was a single-center, retrospective comparative study. This study was approved by the Institutional Review Board of Gachon University Gil Medical Center and followed the tenets of the Declaration of Helsinki. This study included the records of 36 eyes of 36 patients with refractory glaucoma that were unresponsive to medical treatment and underwent implantation of an AGV (New World Medical, Inc., Rancho Cucamonga, CA) by 1 of 2 surgeons (Dae Yeong Lee or Jong Yeon Lee) between March 1, 2008, and June 31, 2012. A model flexible plate FP-7 or FP-8 AGV was used in all 36 eyes. The eyes were divided into two groups according to the surgical methods. Eighteen eyes that underwent the pars plana implantation of an AGV with a concurrent vitrectomy (ppAGV) and 18 eyes that underwent the implantation of an AGV in the anterior chamber (acAGV) were examined.

The inclusion criteria were refractory glaucoma with an intraocular pressure (IOP) ≥ 26 mmHg despite the maximum tolerated oral and topical antiglaucoma medical therapy. Cases with previous cataract surgery and/or vitrectomy were included. Exclusion criteria included a history of corneal disease, previous penetrating keratoplasty, more than one shunt in the same eye, hypotony associated with markedly flat AC, cases with <15 months of follow-up period, cases of concurrent cataract extraction surgery, and cases of previous glaucoma surgery with <1500 endothelial cell counts.

In the ppAGV, AGV implantation was combined with a vitrectomy under retrobulbar anaesthesia. A fornix-based conjunctival flap and a Tenon's capsule dissection with relaxing incisions were made in either the superotemporal or superonasal quadrants. The plate body was then anchored 8–10 mm posterior to the limbus between the rectus muscles. If the subjects had not undergone vitrectomy, a 23-gauge vitrectomy was performed using a DORC two-step system (Dutch Ophthalmic Research Company, Exeter, NH). In all of the cases, meticulous care was taken to shave the vitreous base in the quadrant, where the AGV tube was to be placed. A sclerotomy was placed 3.5 mm posterior to the limbus in all eyes. After the implant tube was trimmed so it would lie at a length of 3-4 mm into the vitreous cavity, the tube was inserted through the cutter probe sclerotomy site and sutured to the episclera with 9-0 nylon. The implant entry site was covered with either a banked sclera or preserved pericardium patch graft. After removing the cannula in the remaining sclerotomy sites, if the wound leakage was observed, a releasable suture was applied with 8-0 nylon followed by closure of the overlying conjunctiva and Tenon's capsule.

In the acAGV, the tip of the drainage tube was cut and then beveled up to extend 3 mm into the anterior chamber. At approximately 1 mm posterior to the surgical limbus, the drainage tube was inserted into the anterior chamber parallel to the iris plane and secured to the sclera with 10-0 nylon sutures. The following procedure was similar to that of ppAGV.

Specular microscopy using a noncontact type specular microscope (Noncon Robo SP-3000P; Konan Medical Inc., Tokyo, Japan) was performed by one experienced examiner. The IOP was measured using Goldmann applanation tonometry by experienced glaucoma specialist (Jong Yeon Lee).

By reviewing the medical records of the 36 cases in detail, we collected preoperative data, including patient age, sex, glaucoma diagnosis, lens status, glaucoma medications, visual acuity, IOP, and corneal endothelial cell count. Postoperative data regarding IOP, visual acuity, and postoperative complications were collected at 1 week and 1, 6, 12, and 18 months after surgery. The postoperative corneal endothelial cell count was checked between 15 and 20 months after surgery.

The postoperative endothelial cell counts, postoperative best corrected visual acuity, and IOP in the 2 groups were compared. Surgical success was defined as a final IOP ≥5 and ≤21 mmHg, with or without additional glaucoma medications, no additional glaucoma surgery, no removal of the implant, and no loss of light perception.

Statistical analysis was performed using SPSS ver. 17.0 (SPSS Inc., Chicago, IL, USA). The data was analyzed using a Mann-Whitney *U* test, Fisher's exact test, and a Wilcoxon signed rank test. *P* value <0.05 was considered significant.

## 3. Results

The mean follow-up period in the ppAGV and acAGV groups was 18.0 (range, 15–20) months and 18.0 (range, 16–20) months, respectively (*P* = 0.78).  Table 1 lists the demographic data of the 2 groups. The age, sex, lens status, previous intravitreal bevacizumab treatment, and glaucoma type were similar in the 2 groups (*P* > 0.05). The preoperative and postoperative data of the 2 groups are shown in Table 2. The mean preoperative corneal endothelial cell count, best corrected visual acuity, IOP, and number of antiglaucoma medications were similar in the 2 groups (*P* > 0.05). At the postoperative 18 months of follow-up, the mean endothelial cell count in the ppAGV and acAGV groups was 2044 ± 303 cells/mm^2^ (95% CI: 1893 to 2196) and 1904 ± 324 cells/mm^2^ (95% CI: 1742 to 2065), respectively, and was not significant between the 2 groups (*P* = 0.25). At the postoperative 18 months of follow-up, the mean IOP decreased significantly from the preoperative values in both groups (*P* < 0.001), but there were no differences in the IOP and number of antiglaucoma medications between the 2 groups (*P* > 0.05). Moreover, the success rate was also not different between 2 groups at 18 months after surgery (*P* > 0.05) (Table 2). The number of endothelial cell losses in the ppAGV and acAGV groups at 18 months after the surgery was 292 ± 120 cells/mm^2^ (95% CI: 232 to 352) and 430 ± 140 cells/mm^2^ (95% CI: 361 to 500), respectively, and showed significant difference between the 2 groups (*P* = 0.005) (Table 3).

**Figure 1:**
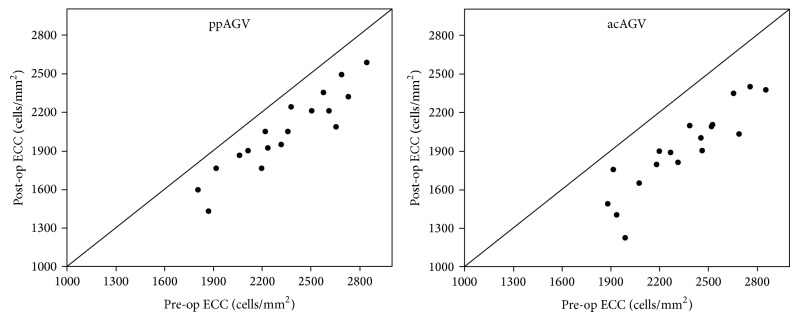
Scatter plots of change for each case in pars plana implantation and anterior chamber implantation of Ahmed glaucoma valve implant.* ppAGV* pars plana implantation of Ahmed glaucoma valve,* acAGV* anterior chamber implantation of Ahmed glaucoma valve, and* ECC* endothelial cell counts.

Figure 1 shows scatter plots of change for each case in both groups. The acAGV was more variable, and decreased endothelial cell counts of acAGV by more than 300 cells/mm^2^ outnumbered ppAGV.

**Figure 2:**
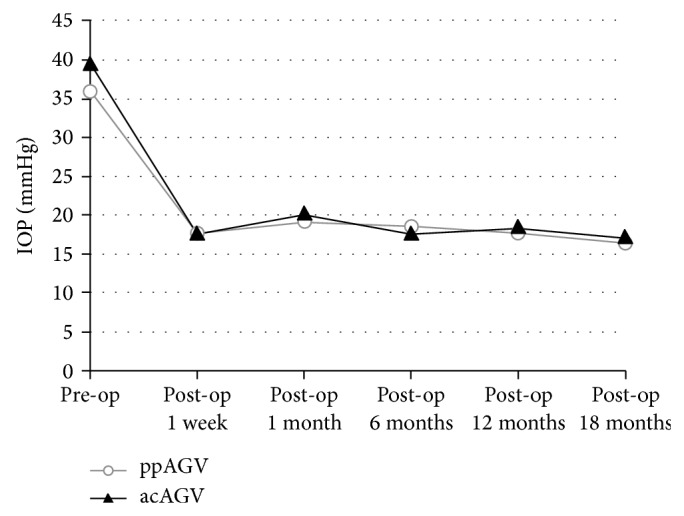
Mean intraocular pressure after in pars plana implantation and anterior chamber implantation of Ahmed glaucoma valve implant.* IOP* intraocular pressure,* ppAGV* pars plana implantation of Ahmed glaucoma valve,* acAGV* anterior chamber implantation of Ahmed glaucoma valve, and* Op* operation.

Figure 2 shows the mean preoperative IOP and the IOP at each of the postoperative times in both groups. At all follow-up times, the mean postoperative IOP was lower than the mean preoperative IOP, and each IOP tended to follow similar patterns in both groups.

All eyes of each group showed favorable visual acuity with an improvement in the mean log MAR visual acuity from 1.76 ± 0.91 preoperatively to 1.27 ± 0.97 postoperatively in the ppAGV and from 2.10 ± 0.93 preoperatively to 1.64 ± 1.18 postoperatively in acAGV, but the differences were not significant (*P* > 0.05).

There were manageable postoperative complications in this study. The incidence of complication is shown in Table 4. Two eyes in the ppAGV group and 1 eye in the acAGV group experienced vitreous haemorrhage after surgery. All vitreous haemorrhages occurred in the eyes with proliferative diabetic retinopathy. More complications such as hyphaema were observed in the acAGV. One eye in the ppAGV group and 3 eyes in the acAGV group showed hyphaema after surgery. The vitreous haemorrhage and hyphaema were stabilized and resolved without management. Overall, no serious complications were observed in both groups after surgery.

The IOP of 2 eyes in the ppAGV group and 1 eye in the acAGV group was elevated despite administration of antiglaucoma medical therapy after surgery. These eyes needed a reoperation. In the ppAGV group, the 2 eyes underwent implantation of an AGV in the anterior chamber. In the acAGV group, the 1 eye underwent a revision of the implants at postoperative 1 month. No cases of corneal touch developed in either group.

## 4. Discussion

A functioning corneal endothelium is essential for corneal integrity and transparency [17]. The corneal endothelium decreases with age but the natural course of endothelial reduction is only 0.6 ± 0.5% per year [18]. On the other hand, when the AGV tube is inserted into the anterior chamber, a reduction of the anterior chamber pressure might lead to an anterior shift of the iris diaphragm, tube-corneal touch, and mechanical trauma to the corneal endothelium [12]. Therefore, the anterior chamber might not be a suitable site for tube implantation in cases, such as corneal diseases, and other anterior chamber abnormalities [19]. The precise mechanism that causes endothelial loss is unclear. McDermott et al. [20] proposed the following as possible mechanisms of corneal endothelial damage: jet flow around the tube end caused by the heartbeat, inflammation in the chamber, intermittent tube-corneal touch, tube-uveal touch, and a foreign body reaction to the silicone tube. On the other hand, pars plana placement of a drainage implant decreases the chance of endothelial loss [17]. When the tube is inserted into the pars plana, the chances of inflammation in the anterior chamber and intermittent tube-corneal touch occurring might have decreased.

Kim et al. [14] reported a 10.5% decrease in the central corneal endothelial cell density at 12 months after AGV implantation in the anterior chamber. Lee et al. [15] reported that the corneal endothelial cell loss after anterior chamber implantation of the AGV was 15.3% and 18.6% at 12 and 24 months after surgery, respectively. Chihara et al. [12] reported that the corneal endothelial cell loss after pars plana implantation of the AGV was 10.2% at 12 months after surgery. Previous study had shown that pars plana implantation of an AGV results in minimal endothelial loss in refractive glaucoma. On the other hand, there are no reports that have compared the loss of corneal endothelial cells after pars plana insertion of an AGV with that after anterior chamber insertion of an AGV.

In this study, we retrospectively examined the corneal endothelial cells after a pars plana and anterior chamber Ahmed valve implant. To our knowledge, this is the first direct comparison between posterior and anterior placement of the Ahmed valve implant. At a mean 18 months of follow-up postoperatively, the ppAGV and acAGV groups showed 12.5% and 18.4% decrease in the number of corneal endothelial cells. This was similar to that reported by Lee et al. [15] in anterior chamber implantation and slightly higher than that reported by Chihara et al. [12]. This reason might be due to the differences in patients' situations, follow-up period, surgical technique, and differences in the methodology.

The cause of endothelial cell loss after shunt surgery is multifactorial and is not fully understood.

Preoperative factors, such as earlier surgeries, episode of chronic inflammation, and the status of endothelial cell before shunt surgery, can all contribute to endothelial cell loss [21]. Eyes in the ppAGV group were more likely to have comorbid retinal disease such as vitreous hemorrhage and tractional membrane at the time of concurrent AGV and PPV surgery. Meanwhile, the acAGV group had more patients with a history of prior filtration surgery, which could influence postoperative endothelial changes. Therefore we excluded cases of previous glaucoma surgery with <1500 endothelial cell counts. Although the preoperative endothelial cell counts were similar between the two groups (*P* = 0.98), we cannot rule out that the preoperative endothelial cell condition may be different and induce the postoperative endothelial changes.

Other causes of endothelial loss may include effects of combined cataract surgery. Endothelial loss following phacoemulsification and IOL implantation under hyaluronate protection is about 3.2–5.9% [22].

In recent study, there was a significant difference between endothelial loss of combined and uncombined cataract surgery in pars plana Ahmed valve implantation [12]. In the cases of combined cataract surgery, cataract surgery itself could decrease the endothelial cell count. Therefore, this study excluded the cases combined with cataract surgery and the cases of previous cataract surgery within 6 months.

Some clinical points should be considered such that the ppAGV might be difficult in phakic eye. In our study, only one case was phakic in the ppAGV whereas 6 cases were phakic in the acAGV. Considering that the injury to endothelial cells may require multiple insults, it is somewhat paradoxical that the preoperative endothelial cell counts were similar between the two groups. Although the difference of the lens status is not significant (*P* = 0.09), it is the limitation to this study such as the relatively small number of patients in the two groups.

Another important cause of endothelial cell loss is high IOP [23]. Postoperative transient ocular hypertension exceeding 25 mmHg has been implicated in endothelial cell damage [12]. In our study, each IOP showed a similar pattern in both groups during the follow-up periods (Figure 2). Until the postoperative last follow-up, 2 cases in the ppAGV group and one case in the acAGV group had an ocular hypertension exceeding 25 mmHg, showing no significant difference between the two groups (*P* = 1.000).

Postoperative inflammation might be implicated in reduction of corneal endothelial cell density [14]. In this study, we did not observe the recurrent iritis which might affect our results.

The postoperative complication, hyphaema, is another possible cause of endothelial cell loss but we observed only 4 cases (ppAGV 1, acAGV 3), which is not significant.

In this study, there is no difference in the preoperative surgery, lens status, glaucoma type, pre- and postoperative IOP changes, surgical outcome, and postoperative complications between the two groups.

Therefore, the location of the Ahmed valve tip could be a major factor affecting the difference in corneal endothelial cell loss.

Our result showed that the difference of the endothelial cell loss between the acAGV and ppAGV at 18 months was 5.9%, which is significant. In acAGV, continued micromotion of the tube relative to the cornea could lead to a continuing low-grade inflammation and to progressive endothelial cell loss [21]. On the other hand, when the tube is inserted into the pars plana, the aqueous humor bypasses the anterior chamber and may lead to anterior ischemia and damage to cornea endothelium. Oh et al. reported that ensuring a sufficient distance from tip of the silicone tube to the cornea is important to minimize the loss of corneal endothelial cell after Ahmed valve implantation [24]. Additionally, we should focus on the shallow or flat AC and hyphaema presents more risk of corneal decompensation in eyes with acAGV than ppAGV.

Thus, the results suggest that the ppAGV might be better than the acAGV in the shunt surgery of the glaucoma patients having low endothelial cell counts.

Previous to this study, we showed that combined 23-gauge vitrectomy and pars plana Ahmed valve implantation was an effective and safe surgery in PDR patients with refractory NVG [19]. Nevertheless, we have to consider the possible posterior segment complication of ppAGV.

Posterior segment complications associated with the ppAGV include retinal detachment, tube obstruction by the vitreous, and placement of the tube into the suprachoroidal or subretinal space [25]. Most posterior segment complications are induced by hypotony, such as decompression retinopathy, choroidal effusion, and hypotony maculopathy [12].

Complete removal of the vitreous at the site of an implant is crucial to the success in pars plana implant surgery to prevent obstructions of the tube of the AGV [25]. In the present study, hypotony-associated complications occurred in only one case in the ppAGV group, which was transitory and might be a cause of leakage in the other sutureless incision site. A previous study reported that the lumen of the Ahmed valve tip fits the 23-gauge needle, which results in no aqueous leakage [19].

This study is limited by the small sample size and retrospective nature, short follow-up period, and variable level of endothelial cell damage at the baseline. Small number of patients made it difficult to interpret the data even though there are one or two cases of severe endothelial cell loss. For example, only one case is 762 cells/mm^2^ of endothelial cell loss in the acAGV, which may influence the results. With a larger patient population, additional studies may provide more accurate results.

This study limitation includes that endothelial cell counts were only counted preoperatively and then once at 18 months. Other investigators reported that postoperative endothelial cell loss increased with time [12, 15]. Lee et al. [15] report that the endothelial loss after anterior chamber implantation of the Ahmed glaucoma valve was 15.3% at 12 months and 18.6% at 24 months. If endothelial cell counts were obtained at several time points such as 6, 12, 18, and 24 months in this study, it is possible to make data interpretation clear. Our study needs to be undertaken in analysis of several time postoperative endothelial cell counts in the future.

Although both the case and control groups were matched one-to-one for glaucoma type, we did not match patients on the basis of age, sex, underlying disease, or severity of glaucomatous disease. We believe that age, especially, and also the other parameters are important factors that influence the loss of corneal endothelial cells even though there is no significant difference in this study.

Therefore, further prospective and randomized trials with a longer follow-up period will be needed.

In summary, corneal endothelial cell damage in the ppAGV group for refractory glaucoma appeared lower than that in the acAGV group. Therefore, pars plana implantation of AGV may be preferred as it may have lower level of endothelial cell damage while maintaining similar level of IOP control.

## Figures and Tables

**Table 1:** Patient demographics.

Characteristics	ppAGV	acAGV	*P* value
Number of eyes (number of patients)	18 (18)	18 (18)	
Mean follow-up periods (months) (range)	18.0 (15~20)	18.0 (16~20)	0.78
Age (y, mean ± SD)	52.9 ± 15.7	53.4 ± 17.8	0.69
Sex, male : female	9 : 9	8 : 10	0.80
Lens status, phakic : pseudophakic	1 : 17	6 : 12	0.09
Previous IVBe	6	5	0.49
Glaucoma type			1.00
Neovascular	16	16
Chronic angle-closure	1	1
Uveitic	1	1

ppAGV: pars plana implantation of an Ahmed glaucoma valve (AGV), acAGV: anterior chamber implantation of an AGV, and IVBe: intravitreal bevacizumab injection.

**Table 2:** Preoperative and postoperative 18-month values in the ppAGV and acAGV groups.

	ppAGV (*n* = 18)	acAGV (*n* = 18)	*P* value
ECC, cells/mm^2^			
Preoperative	2337 ± 311	2334 ± 302	0.98
Postoperative	2044 ± 303	1904 ± 324	0.25
*P* value	<0.001	<0.001	
BCVA, LogMAR			
Preoperative	1.76 ± 0.91	2.10 ± 0.93	0.31
Postoperative	1.27 ± 0.97	1.64 ± 1.18	0.21
*P* value	0.070	0.063	
IOP, mmHg			
Preoperative	35.9 ± 7.6	39.7 ± 12.7	0.26
Postoperative	16.5 ± 7.5	17.1 ± 8.6	0.87
*P* value	<0.001	<0.001	
Glaucoma medication			
Preoperative	2.9 ± 0.3	2.7 ± 0.6	0.39
Postoperative	1.5 ± 0.8	1.6 ± 0.8	0.52
*P* value	<0.001	<0.001	
Surgical outcome (%)			
Success	16 88.8	14 77.8	0.66
Failure	2 11.2	4 22.2	

ppAGV: pars plana implantation of an Ahmed glaucoma valve (AGV), acAGV: anterior chamber implantation of an AGV, ECC: endothelial cell count, BCVA: best corrected visual acuity, LogMAR: logarithm of the minimal angle of resolution, and IOP: intraocular pressure.

**Table 3:** Comparison of endothelial cell loss at postoperative 18 months between ppAGV and acAGV groups.

	Preoperative ECC, cells/mm^2^	Decreased ECC, cells/mm^2^	% of loss
Total (range)	2336 ± 302 (1805–2854)	361 ± 135 (135–762)	15.5
ppAGV (range)	2337 ± 311 (1805–2842)	292 ± 120 (135–571)	12.5
acAGV (range)	2334 ± 302 (1879–2854)	430 ± 140 (158–762)	18.4
*P *value (ppAGV versus acAGV)	0.98	0.05	NA

ppAGV: pars plana implantation of an Ahmed glaucoma valve (AGV), acAGV: anterior chamber implantation of an AGV, ECC: endothelial cell count, and NA: not applicable.

**Table 4:** Postoperative complications in the ppAGV and acAGV groups.

Complications	ppAGV (*n* = 18)	acAGV (*n* = 18)
Vitreous hemorrhage	2	1
Hyphaema	1	3
Elevated IOP (>25 mmHg)	2	1
Transient hypotony (<5 mmHg)	1	0

ppAGV: pars plana implantation of an Ahmed glaucoma valve (AGV), acAGV: anterior chamber implantation of an AGV, and IOP: intraocular pressure.
